# Preliminary Notes on a Case of Abdominal Aneurism

**Published:** 1901-09

**Authors:** Marshall Clinton

**Affiliations:** Buffalo, N. Y.; Attending surgeon, Erie County Hospital; assistant attending surgeon, Sisters of Charity Hospital and Buffalo Children’s Hospital; surgeon Pennsylvania R. R., etc.


### CLINICAL REPORT.

### Preliminary Notes on a Case of Abdominal Aneurism.

By MARSHALL CLINTON, M. D., Buffalo, N. Y.,

Attending surgeon, Erie County Hospital; assistant attending surgeon, Sisters of Charity Hospital and Buffalo Children's Hospital; surgeon Pennsylvania R. R., etc.

W. H. G., aged 57; U. S.; carpenter; married. Admitted Erie County Hospital, July 8, 1901.

*Family History.*—Father died at the age of 65 of cancer of the face; mother died of congestion of lungs. Two brothers and five sisters. One sister has female trouble, one died of consumption, one of heart disease, the rest all well and healthy. Has two children, both boys—one 19 years old at Craig Colony for Epileptics, the other alive and well.

*Previous History.*—Never has used alcohol or tobacco, has always worked hard at his trade, doing some painting occasionally. Has had measles and whooping cough when a child. Thirty years ago used to have attacks of vomiting at night after eating heavily. Four and five years ago had frequent attacks of jaundice which would be relieved by doses of Rochelle salts. Had dizzy spells frequently, fifteen years ago, and headaches at night. Two years ago was bothered with palpitation on exertion.

*Present illness.*—Four years ago was lifting a heavy piece of lumber where he felt something give way in his belly. Had considerable pain that night and the next day and managed to go to work again, working for six to eight weeks. One morning before rising he noticed as he passed his hand over his belly that he felt a lump on the right side. After he stood up and passed his urine the lump disappeared, so he put it out of his mind and did not notice it until in bed some nights later.

In the fall of 1900, was walking three miles to his home after a hard day's work, when he felt something give out in his belly and a severe pain followed. He sat down by the roadside and rested for fifteen minutes and the pain disappeared. As soon as he began to try to walk the pain would come back, so he was forced to take short walks and long rests, and in this way reached home. The pain stopped when he went to bed, and he got up the next morning apparently all right. Did light work for two weeks, when the pain returned and became a dull heavy ache while on his feet disappearing when he is in bed. On advice of his physician he came to Buffalo to be operated on for an enlarged gall bladder.

*Examination.*—Patient fairly nourished, and slightly jaundiced; temperature subnormal; walks with difficulty; pupils equal and react to light and distance; slightly deaf; tongue, slightly coated; heart, normal; lungs, normal; feet cold and slightly numb; pulse, 74; respiration, normal; abdomen shows a bulging mass in the right hypochondriac region, dome-shaped, four by two inches wide and cystic in feel. No pulsation can be seen or felt and no thrill or bruit can be heard over the mass or over the femorals.

*Diagnosis.*—Stenosis of the cystic duct and retention cyst of the gall bladder.

*Operation*, July 25, 1901.—An incision, four inches long, parallel with the border of the ribs and over the prominent part of the tumor down to the peritoneum revealed a dark almost black tumor adherent to the peritoneum. As the color was so unlike a gall bladder an aspirating needle was plunged into the center of the mass and was followed by a gush of bright, red blood through the lumen of the needle. Adhesions along the lower border were separated to permit of better inspection and after the lower edge was freed, it was seen to have a distinct fibrous capsule and to pulsate. The needle introduced an inch and a half from the first point of entrance was followed by blood, as in the first trial. A patient effort was made to introduce silver wire through the needle, but the available wire was unsuitable in size and would kink as soon as the end struck the opposite side of the sac. The openings made by the needle were closed by holding the finger lightly over them for a few minutes, when a clot would form under the finger and the hole be plugged. It was possible to introduce the finger along the lower border of the sac in the direction of the abdominal aorta.

I believe it to be an aneurism of this vessel from the fact that my finger passed to the aorta, and that the aneurism is pressing on the anterior spinal roots, causing pain and difficult walking. With the patient's consent, further efforts will be made to introduce suitable wire and run a low galvanic current through the wire. This case is of interest to any surgeon from the fact that the lesion, though rare and unsuspected before operation, was detected from the fact that the dark color threw doubt into the operator's mind as to the nature of the mass.

466 Franklin Street.

